# Paraneoplastic Pemphigus: Insight into the Autoimmune Pathogenesis, Clinical Features and Therapy

**DOI:** 10.3390/ijms18122532

**Published:** 2017-11-26

**Authors:** Giovanni Paolino, Dario Didona, Giuseppe Magliulo, Giannicola Iannella, Biagio Didona, Santo Raffaele Mercuri, Elisa Moliterni, Michele Donati, Andrea Ciofalo, Guido Granata, Patricia Ranuzzi, Vincenzo Falasca, Stefano Calvieri

**Affiliations:** 1 Unit of Dermatology and Cosmetology, IRCCS University Vita-Salute San Raffaele, 20132 Milan, Italy; mercuri.santoraffaele@hsr.it; 2 Dipartimento di Medicina Interna e Specialità Mediche, Dermatologia, Sapienza Università di Roma, Piazzale Aldo Moro, 5, 00185 Roma, Italy; elisa.moliterni@gmail.com (E.M.); stefano.calvieri@uniroma1.it (S.C.); 3 Prima Divisione di Dermatologia, Istituto Dermopatico dell’Immacolata-IRCCS, Via dei Monti di Creta, 104, 00167 Roma, Italy; dario.didona@gmail.com (D.D.); b.didona@idi.it (B.D.); 4 Dipartimento di Organi di Senso, Sapienza Università di Roma, Piazzale Aldo Moro, 5, 00185 Roma, Italy; giuseppe.magliulo@uniroma1.it (G.M.); giannicola.iannella@uniroma1.it (G.I); andrea.ciofalo@uniroma1.it (A.C.); vincenzo.falasca@uniroma1.it (V.F.); 5 Dipartimento di Anatomia Patologica, Università Campus-Biomedico, 00128 Roma, Italy; micheledonati25@gmail.com; 6 Dipartimento di Immunologia Clinica, Sapienza Università di Roma, Piazzale Aldo Moro, 5, 00185 Roma, Italy; guido.granata@uniroma1.it; 7 Dipartimento di Farmacologia, Sapienza Università di Roma, Piazzale Aldo Moro, 5, 00185 Roma, Italy; patricia.ranuzzi@alice.it

**Keywords:** oncology, oral lesions, paraneoplastic pemphigus, therapy

## Abstract

Paraneoplastic pemphigus is a rare autoimmune skin disease that is always associated with a neoplasm. Usually, oral, skin, and mucosal lesions are the earliest manifestations shown by paraneoplastic pemphigus patients. The pathogenesis of paraneoplastic pemphigus is not yet completely understood, although some immunological aspects have been recently clarified. Because of its rarity, several diagnostic criteria have been proposed. Besides, several diagnostic procedures have been used for the diagnosis, including indirect immunofluorescence, direct immunofluorescence, and ELISA. We reviewed the most recent literature, searching on PubMed “paraneoplastic pemphigus”. We included also papers in French, German, and Spanish. We found 613 papers for “paraneoplastic pemphigus”. Among them, 169 were review papers. Because of its varying clinical features, paraneoplastic pemphigus still represents a challenge for clinicians. Furthermore, diagnosis and management of paraneoplastic pemphigus requires close collaboration between physicians, including dermatologist, oncologist, and otorhinolaryngologist.

## 1. Introduction

Paraneoplastic pemphigus (PNP) was first reported by Anhalt in 1990 [1]. It is a rare autoimmune skin disease belonging to the group of blistering diseases. PNP is always characterized by an association with neoplasms, including carcinoma of the stomach, lung, and colon [2]. Besides, B-cell lymphomas and hematological malignancies are most frequently reported in association with PNP [3,4]. In 2001 Nguyen et al. introduced the concept of paraneoplastic autoimmune multiorgan syndrome (PAMS), highlighting the systemic nature of PNP [5]. In this regard, it should be noted that these patients usually show a multi-organs involvement and different subsets of auto-antibodies to several tissues [1,3,6]. PNP shows a mortality rate up to 90%, and its early diagnosis is not simple [6,7]. Therefore, every effort must be made to diagnose PNP as earliest as possible [1,2,5].

## 2. Epidemiology

Specific data regarding the incidence of PNP are still not available, but it is considered as a rare disease. Indeed, around 500 cases of PNP have been reported in the literature [8,9]. PNP accounts for 3–5% of all pemphigus cases [8,9,10]. It arises usually in patients aged between 45 and 70 years, without any significant difference between male and female [3]. Furthermore, PNP can affect also children and adolescents [11,12,13,14]. In this sub-group of patients, PNP is more frequently associated with Castleman’s disease and hematologic malignant disorders [11,13].

## 3. Etiology

Up to 84% of all PNP cases are caused by hematologic neoplasms or disorders [3,9]. Lymphoproliferative disorders are the most frequent diseases associated with PNP [9]. Non-Hodgkin’s lymphoma is the most common associated neoplasm (38.6%), followed by chronic lymphocytic leukemia (18.4%), Castleman’s disease (18.4%), thymoma (5.5%), Waldenstrom’s macroglobulinemia (1.2%), Hodgkin’s lymphoma (0.6%), and monoclonal gammopathy (0.6%) [3,9,15,16]. In addition, carcinomas from epithelial cells (8.6%) [17,18,19] and sarcomas from mesenchymal lines (6.2%) [12,20,21,22] have been described in association to PNP. Gastric cancers have been rarely reported as a PNP trigger. Indeed, only three cases of PNP related to gastric cancers (1 gastric lymphoma and two adenocarcinomas) have been reported in the literature [23,24]. A single PNP case caused by melanoma, described as localized paraneoplastic pemphigus, has been reported [25]. However, Anhalt criticized this report, because of lacking anti-desmoglein antibodies and clinical features of PNP [26]. Some PNP cases triggered by certain drugs, including fludarabin and bendamustine, have also been described [26,27,28]. However, according to Anhalt [26], the number of drug-linked-PNP could be underrated, because of the absence of a registry for comprehensive data acquisition regarding PNP. Finally, a single PNP case associated with radiotherapy has been described [29].

## 4. Genetic

Recently, it has been reported that PNP was associated with the DRB1*03 allele and HLA-Cw*14 alleles. These genetic characteristics are more frequent in some PNP populations, such as Caucasian and Chinese ones [30,31]. These conclusions were drawn respectively from a series of 13 Caucasian French patients by Martel et al. [30] and of 19 Han Chinese patients by Liu et al. [31].

## 5. Pathogenesis

Although the pathogenesis of PNP is still not completely known, it is plausible that both autoantibodies and cell-mediated immunity play a key role [1,3,4,5]. The most common auto-antibodies detected in PNP were directed against the plakin family, including antibodies against the 210-kDa envoplakin, the 190-kDa periplakin, the 250- and 210-kDa desmoplakins I and II, the 500-kDa plectin, and the 230-kDa bullous pemphigoid (BP) antigen [32,33,34,35].

Antibodies directed against plakophilin 3 and desmocollins 1–3 have also been reported in different papers [36,37]. Furthermore, it has been thought that autoantibodies against desmoglein-1 (DSG-1) and desmoglein-3 (DSG-3) might also play a pathogenic role in PNP [38,39], although Amagai et al. found a 100% positivity only for anti-DSG-3 autoantibodies [38]. The protease inhibitor A2-macroglobulin-like-1 (A2ML1) has been also thought to be a pathogenic element in PNP [40,41]. Besides, recently, Tsuchisaka et al. reported that epiplakin was a PNP autoantigen [42]. In that paper, epiplakin was detected in 72.9% of 48 PNP sera of Japanese patients by immunoprecipitation-immunoblotting [42]. In addition, the authors showed that epiplakin-negative PNP cases did not develop bronchiolitis obliterans. Indeed, epiplakinis is reported as a target antigen in PNP-related bronchiolitis obliterans [42].

PNP antibodies belong principally to the IgG class. However, IgA class has also been reported in few cases [43,44,45,46]. More specifically, Mentink et al. detected by ELISA IgA autoantibodies only against DSG-3 in a series of four PNP patients [47]. Interestingly, in only one patient the serum showed IgA, but not IgG autoantibodies [43].

Cell-mediated immunity could also play a role in PNP [3,48]. Reich et al. found selective epidermal activated CD8+ T-cells in PNP [49]. In addition, Cummins et al. reported some PNP patients without any detectable autoantibodies [50]. Furthermore, Wade et al. detected MHC-restricted CD8+ cytotoxic T cells, non-MHC-restricted CD56+, and CD68+ natural killer cells within the dermo-epidermal junction of PNP lesions [51].

## 6. Clinical Features

PNP clinical features are extremely polymorphous and PNP lesions can be detected not only on the skin, but also in different mucosae [1,2,3,11]. The presence of different autoantibodies could justify the different clinical features in PNP patients [3].

Usually, a neoplasm is detected before the onset of PNP [1,8,52]. However, PNP is the first clinical manifestation that leads to the detection of an occult tumor in about 30% of cases [3,9].

Usually, oral and skin lesions are the earliest manifestations [52,53,54].

Because the refractoriness of the oral mucosal and skin lesions to standard immunosuppression therapy, patient should be screened for an underlying occult neoplasm [1,8]. Because of the lack of standardization of a screening protocol for occult neoplasms, a pan-CT scan and flow cytometry on peripheral blood should be mandatory; indeed, PNP is mostly associated to lymphoproliferative or haematological neoplasms [1,8].

### 6.1. Oral Lesions

**Figure 1:**
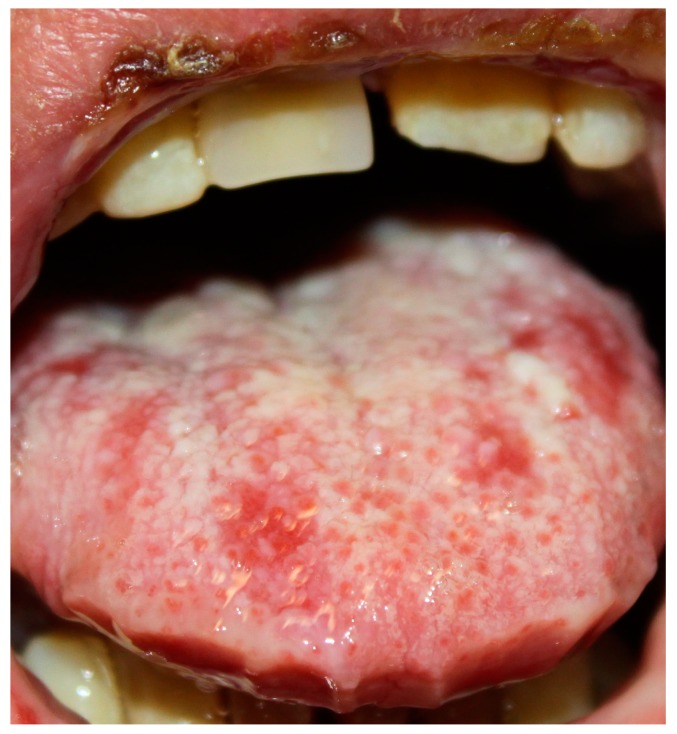
Paraneoplastic pemphigus (PNP) oral lesions. Ulceration involve oral mucosal surface and tongue. Stomatitis is also visible.

**Figure 2:**
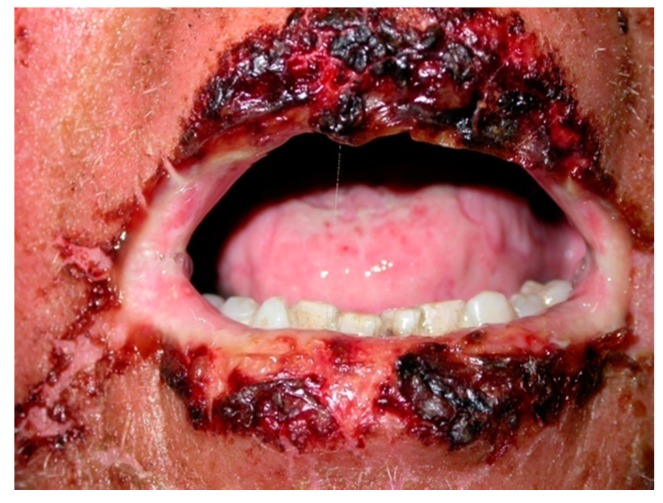
PNP peri-oral lesions. Ulceration with crusting over the peri-oral region and lips.

Oral mucosa is almost always involved [2,21]. Clinically, PNP often presents with oral erosions, which are rarely preceded by vesicles or bulla. The lesions usually involve the vermillion border of the lips (Figure 1 and Figure 2). Ulceration may involve the entire oral mucosal surfaces and may represent the sole manifestation of this condition. A painful stomatitis is also commonly shown caused by massive erosion in the oropharynx [2,21,53,55].

Healy WJ et al. reported the case of a 51-year-old man who was admitted to the emergency department with a 2–3-month history of dyspnea as well as oral ulcerations [55]. The oral ulcerations were biopsied, showing PNP features [55]. In addition, Mahajan et al. described a case of a 58-year-old female with a one-year history of recurrent, multiple flaccid bullae and erosions with oozing, crusting, and severe painful oral ulcerations [56]. Several treatments, including repeated courses of systemic corticosteroids, provided temporary relief, but recurrences and exacerbations caused marked discomfort and disability. The patient had severe erosions/ulceration of the vermilion lips, palate and tongue, and conjunctival congestion (conjunctivitis). The oral lesions preceded the skin lesion appearance by two months.

It should be also noted that extended, painful erosions and crusting on the lips could resemble oral lesions commonly found in erythema multiforme (EM) or Stevens-Johnson’s disease [2,21,53].

In children, the stomatitis caused by PNP may be often mistaken for herpetic stomatitis or toxic epidermal necrolysis (TEN), leading to a delay in the diagnosis [57]. In this subset of patients, the most important cause of PNP is Castelman’s disease [58].

The role of cell-mediated immunological mechanisms in PNP epithelial injury have been examined [3,4,5]. Infiltrates in the oral epithelia consist mainly of CD8+ T cells and CD68+ macrophages, mixed with CD56+ natural killer cells [1,5,55,59,60].

### 6.2. Secondary Mucosal Lesions

The nasopharynx, ano-genital region, and esophagus could be also affected by large, painful, mucosal lesions [1,3,9,15,59,60]. Yokokura et al. reported the case of a 58-year-old Japanese man with an eight-month history of painful lesions on the lower lip, oral mucosa, and genital region [61]. Physical examination revealed well-demarcated erosion with grayish hyperkeratosis and violet-reddish erythema at the border on the lower lip and glans penis. Multiple erosions were also distributed from the hard palate to the pharynx. 

Conjunctival lesions could be also present in PNP patients. Meyers SJ et al. reported two cases of bilateral bulbar conjunctival hyperemia and diffuse papillary tarsal conjunctival reactions [59]. One patient had sloughing of the conjunctival epithelium, and the other ones had tarsal conjunctival scarring and forniceal shortening. Histopathological findings from conjunctivae samples from both patients were consistent with pemphigus vulgaris (PV).

Ocular involvement occurs in 70% of PNP patients. Several different ocular symptoms have been reported, including painful ocular irritation, worsening of vision, and mucus discharge [5,59,62]. Clinical signs can include conjunctival erosions, eyelid margin thickening, corneal erosions, and pseudomembranous conjunctivitis [62].

### 6.3. Skin Lesions

**Figure 3:**
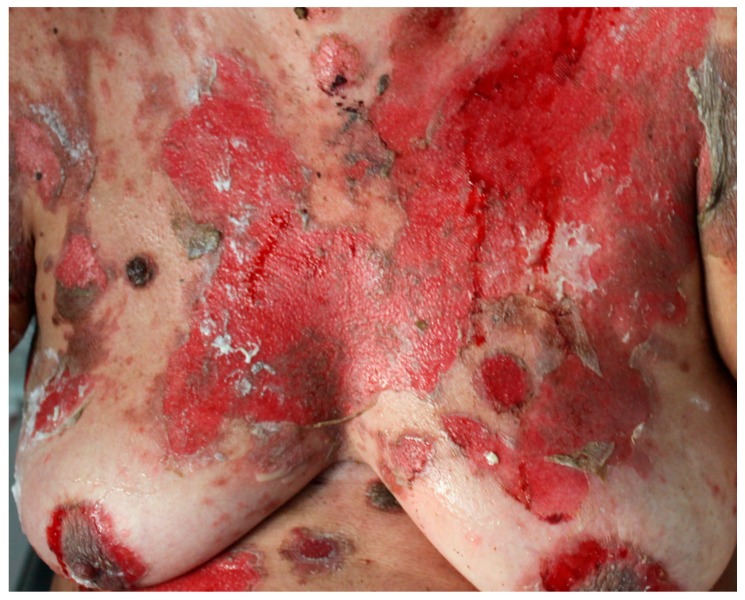
PNP skin lesions. Diffuse erythema, exfoliative erythroderma, erosions, or ulcerations on the trunk and abdomen.

**Figure 4:**
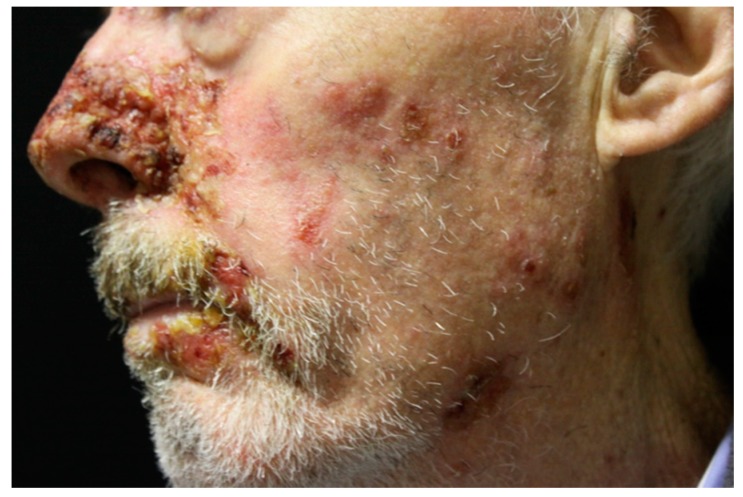
PNP head and neck lesions. Erythema and ulceration with oozing and crusting.

Usually, skin lesions appear after the onset of mucosal lesions [3,52]. Most PNP patients show widespread cutaneous involvement, especially on the torso (Figure 3), head (Figure 4), neck, and proximal extremities [7,63]. PNP onset is highly variable. Patients may present with diffuse erythema, vesiculo-bullous lesions, papules, scaly plaques, exfoliative erythroderma, erosions or ulcerations. The erythema can be macular, urticarial, targetoid or polymorphous. Patients may initially show erythema, and then develop bullae and erosions [5,10,15,16].

A single patient may present different types of lesions, each of which is able to evolve from one type to another [8,52]. Usually, cutaneous lesions resemble those seen in PV, BP, EM, or graft versus host disease (GVHD) [16]. Furthermore, pustular and psoriasiform lesions have also been reported in the literature [5]. 

The different clinical features could be due to the predominance of the cell-mediated or humoral-mediated pathogenic mechanism [51]. On the one hand, humoral-mediated cytotoxicity usually causes a prominent PV-like appearance [3,5,52]; on the other hand, cell-mediated cytotoxicity often determines lichenoid lesions [48,50]. Lichenoid lesions are frequently identified in children, predominantly on the torso and limbs [12,13]. Furthermore, a unique case of pemphigus vegetans-like PNP has been described [64,65].

Pediatric patients often show lichenoid PNP lesions rather than bullous skin lesions [57,66]. In a series of 14 PNP pediatric patients, it has been reported that all of them demonstrated antibodies to plakins [12].

### 6.4. Pulmonary Manifestations

PNP affects also the respiratory epithelium in up to 92.8% of cases [11,51], leading to dyspnea, obstructive lung disease, and bronchiolitis obliterans, which is one of the principal causes of death in PNP patients [9,67]. Pulmonary involvement is commoner in children and Chinese patients with Castelman’s disease [67]. Recently, Tsuchisaka et al. reported a correlation between epiplakin and bronchiolitis obliterans in Japanese PNP patients [42]. Furthermore, the authors reported that 71% of patients were affected by bronchiolitis obliterans pneumonia that led to a worse prognosis despite treatment of the underlying malignancy [12,42,57].

## 7. Pathology

**Figure 5:**
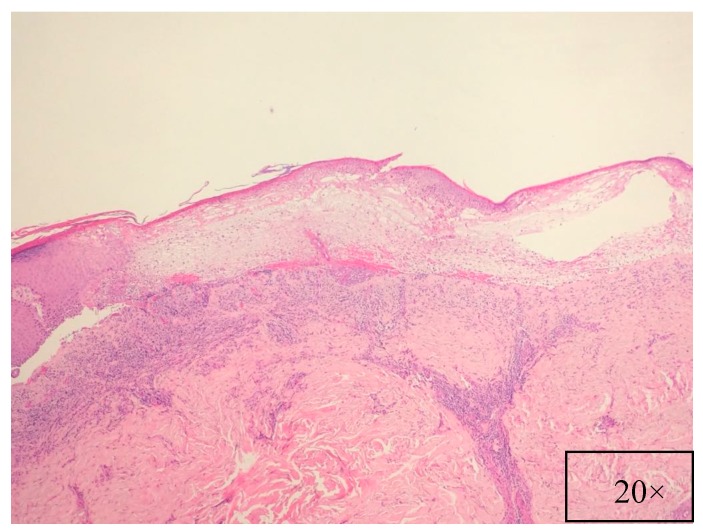
Intense, band-like inflammatory infiltrate, with minimal supra-basal acantholysis (H&E, magnification 20×).

**Figure 6:**
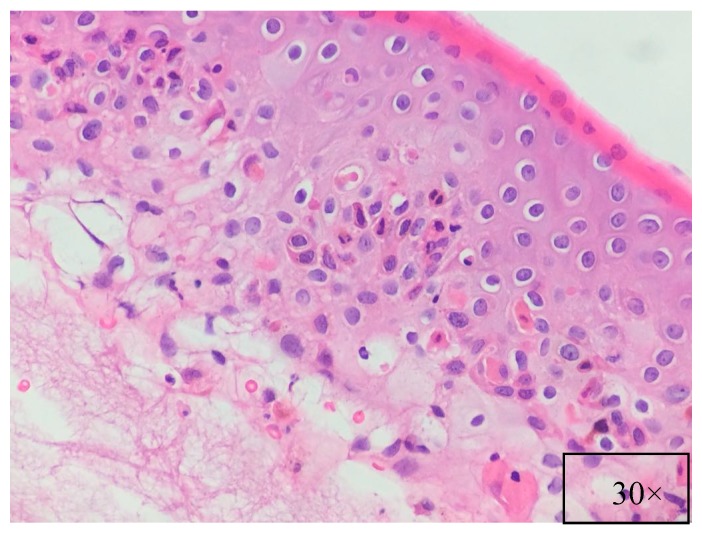
Basal cell vacuolar degeneration, dyskeratotic and necrotic keratinocytes, lymphocytic inflammation with lymphocytic exocytosis. (H&E, magnification 30×).

**Figure 7:**
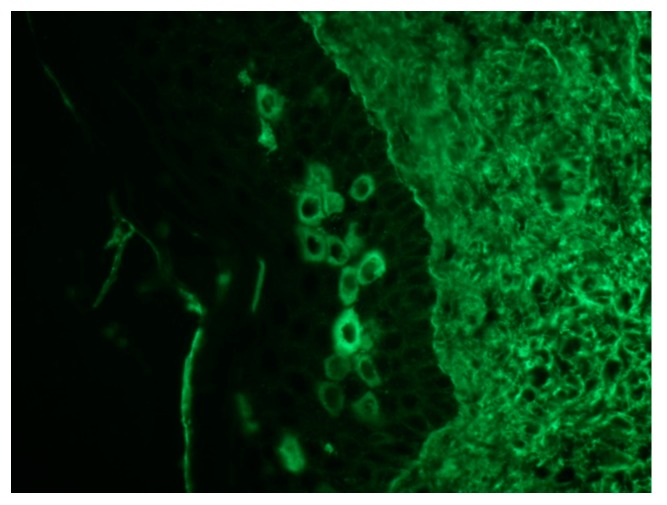
Direct immunofluorescence shows IgG deposition in the epidermal intercellular spaces and in the basement membrane (20×).

Pathological findings are related to the clinical features, showing different pathological characteristics according to the examined lesion [7,39]. When blisters are present, suprabasal acantholysis with sparse inflammatory infiltrates is usually evident on skin biopsy (Figure 5) [39], while interface and lichenoid dermatitis are usually detected if inflammatory maculopapular lesions are present [39,50]. In addition, lesions with mixed clinical features might show both acantholysis and lichenoid interface dermatitis [8,53,54]. Dyskeratosis with suprabasal acantholysis is an important finding that leads to the diagnosis of PNP [8]. (Figure 6) However, sometimes the acantholysis is difficult to find, leading to important diagnostic pitfalls with other diseases as erythema multiforme, Stevens–Johnson syndrome, GVHD, and drug reactions. Finally, it is important to highlight that pathological findings could be compatible with a diagnosis of PNP even when the results of direct immunofluorescence (DIF) are negative [8,53]. Indeed, DIF findings are important because the cells involved in PNP include autoantibodies and CD8+ T cells that attack proteins in different layers of the keratin, leading to different DIF features, including intercellular cement substance staining and/or dermo-epidermal junction staining [8,53] (Figure 7).

## 8. Immunological Studies

Because PNP represents a pitfall for clinicians, several immunological studies have been developed to better detect different PNP antibodies. In this context, PNP patients show plenty of auto-antibodies, targeting Dsg 3, desmoplakin 1 and 2, envoplakin, periplakin, plectin, BP antigen 230 (BP230), and α-2-macroglobulin-like-1, a 170 kd protease inhibitor found in stratified epithelia and other tissue damaged by PNP, which has been recently identified [16,68].

ELISA is a useful tool to detect anti-Dsg 3 and anti-Dsg 1 auto-antibodies in PNP, although PNP patients usually show only anti-Dsg3 IgG [68]. However, PNP patients lacking anti-Dsg autoantibodies have been also described [68]. In 2009, Probst et al. created a new ELISA based on the recombinant N-terminus of envoplakin, showing a sensitivity of 82% and a specificity of ≥98% [69]. However, it has been reported that ELISA appeared to lack sensitivity in a multi-assay comparison across different assays, because antigen-specific techniques, such as envoplakin ELISA, detect only one of a range of autoantibodies potentially responsible for the disease [70].

Immunofluorescence (IF) is one of the main diagnostic tools for PNP. DIF usually shows IgG and/or C3 deposition in the epidermal intercellular spaces (EIS) alone [71]. The presence of IgG and/or C3 in EIS and at the basement membrane zone (BMZ) has been found in less than 50% of cases [71]. Linear deposits of IgG and/or C3 at the BMZ may also be detected [39]. (Figure 7) This pattern could help to differentiate PNP from other forms of pemphigus that show Ig deposits only between keratinocytes [5]. However, DIF is negative in around 50% of cases [71]. False negatives are often due to necrotic tissue (especially in mucosal specimens) and to lichenoid lesions [50,71]. Different substrates could be used to perform indirect immunofluorescence (IIF), including normal human skin, monkey esophagus, rat bladder, rat myocardium, and rat lung [71]. IIF identifies autoantibodies directed against plakins, among which autoantibodies to envoplakin and periplakin are the most specific [39]. On the one hand, IIF on normal human skin has been reported as positive in up to 50%; on the other hand, IIF on rat bladder urothelium has been found positive in 75% of cases, displaying a better sensitivity [72]. In addition, IIF on rat bladder has a high specificity (83%) [1,72]. Therefore, IIF on rat bladder is now thought as a useful screening test for PNP. However, autoantibodies directed against plakins have been also shown in other dermatoses, including PV, pemphigus foliaceus, and TEN [72,73,74].

Immunoprecipitation (IP) is considered the gold standard for diagnosing PNP [75]. IP can show antibodies against several antigens, including plakins and α-2-macroglobulin-like-1 [40]. In addition, a positive IP has been reported by Camisa et al. as a major criterion for diagnosing PNP [76].

Immunoblotting (IB) could be used to detect antibodies against desmoplakin 1 and 2, periplakin, and envoplakin on normal human keratinocytes extract [39,71].

## 9. Diagnosis

According to Anahlt et al. [1] the diagnostic criteria include five different points (Table 1). Subsequently, Camisa et al. [76] introduced different criteria, including major and minor ones. According to Camisa et al., three major or two major and two minor criteria are needed to diagnose PNP [76]. More recently, Mimouni et al. [12] revised the original criteria by Anahlt et al. In this new classification DIF was considered as a non-essential criterion for diagnosing PNP, because of its low sensibility [71,72]. IIF on rat bladder urothelium and monkey esophagus are considered useful in screening for detecting PNP [71,72]. A scheme depicting the diagnostic algorithm is shown in Table 2.

## 10. Differential Diagnosis

Differential diagnosis includes PV, BP, and EM. A more complete list is shown in Table 3. Other diseases with mucous skin involvement should always be excluded [77,78]. Clinically, PNP and PV may share many features, but PNP shows blisters developing from inflammatory papules or macules, while PV usually shows blisters on the erythematous background. In addition, PNP could show antibodies anti-A2ML1, anti-envoplakin, and anti-periplakin, highly specific for PNP [71,72,73,74]. Furthermore, DIF shows epithelial cell-surface IgG depositions with concurrent basement membrane zone IgG depositions, considered a hallmark feature of PNP [71,72]. Finally, unlike other autoimmune blistering diseases, PNP antibodies stain rat bladder epithelium. BP also shares some features with PNP, especially when BP230 and BP180 are detected in PNP. However, DIF in PNP could show the presence of epidermal intercellular deposits of IgG and complement C3, which are not found in BP. EM-like lesions resembling TEN could also be present in PNP, but the detection of auto-antibodies leads to PNP diagnosis [1,2,3,71,72,73,74].

It is important to highlight that oral and cutaneous PNP lesions can be variable and resemble many other diseases, both clinically and histologically. Therefore, an otolaryngologic examination is essential for the evaluation of the lesions and of the differential diagnosis with other oral cavity pathologies [2,21,53,55].

In patients with oral lesions, histologic findings, cutaneous lesions, and IIF support the diagnosis of PNP [21,53,55].

## 11. Treatment Options

PNP therapy remains challenging because of the rarity of the disease. Although several medical therapies have been suggested in the literature, PNP has been considered as more resistant to medical therapies in comparison to other forms of pemphigus [16,79]. When PNP is suspected, it could be useful to follow the six steps reported by Frew et al. for better management of the patient [80]. This series of steps includes stabilization of vital parameters, evaluation of any underlying malignancy, accurate diagnosis of PNP, removal and medical therapy of the trigger tumor, and treatment of PNP using immunosuppression, immunomodulation, or plasmapheresis. The first step is of capital importance because of the high rate of mortality. Therefore, the stabilization of the patient represents the first measure in the management of PNP patients [80].

High-dose corticosteroids are still considered as first line therapy [81,82]. However, steroids only improve the skin lesion, while mucosal involvement it is not usually affected by steroids [54,83]. Indeed, one of the most important clinical features of PNP is the resistance of the mucosal lesions to most types of therapy [83]. Nevertheless, high-dose prednisolone is still recommended as the first line of treatment [80].

It has been reported that the association between prednisolone and other drugs, including azathioprine, cyclosporine, mycophenolate mofetil, cyclophosphamide, intravenous immunoglobulin (IVIG), and plasmapheresis show a good profile of efficacy and safety in selected patients [1,54,81,84,85,86,87,88]. However, mucosal lesions are usually also resistant to combination therapy regimen [83].

Rituximab, an anti-CD20 monoclonal antibody, has been reported as effective in PNP patients caused by B-cell lymphoma [89,90]. Several rituximab schedules have been reported in the literature, including monotherapy (375 mg/m^2^ weekly for four weeks) followed by eight weekly infusions and weekly infusions for four weeks under corticosteroids and other immunosuppressive drugs, such as cyclosporine A [80].

It has been reported that alemtuzumab, a humanized monoclonal antibody that binds CD52, induced long-term remission in a patient with B-cell chronic lymphocytic leukemia [91]. This treatment was used in a patient refractory to many of previous treatment, including corticosteroids, cyclosporine, and IVIG. Alemtuzumab was administered 30 mg intravenously three times a week for 12 weeks, showing improvement of mucosal and cutaneous lesions. Twelve months later, the patient was still in remission on maintenance therapy (500 mg mycophenolate mofetil and 5 mg prednisone) [80,91].

Daclizumab, a humanized monoclonal antibody against the alpha subunit of the IL-2 receptor of T cells, is thought to be a promising therapy for PNP [54].

Early antimicrobial therapy is recommended, because of the risk of sepsis following loss of skin integrity and iatrogenic immunosuppression [16]. Antalgic therapy could be useful in reducing the pain caused by extensive erosions [16].

## 12. Prognosis

The prognosis of PNP is generally poor (90% mortality rate) [8,16]. Death is usually due to systemic complications, including sepsis, gastrointestinal bleeding, and bronchiolitis obliterans [8,16]. It has been reported that PNP and underlying malignancy do not have a parallel evolution [3,8,16]. Indeed, PNP lesions generally progress after removing the triggering malignancy or even when the malignancy is under control [11,13,16]. However, it has been shown that outcome is better in PNP patients with concurrent Castleman’s disease or benign thymomas after removing the tumor [92].

Finally, the prognosis of PNP depends on an appropriate management, including the effective control of the oral and skin lesions, an adequate treatment of the underlying neoplasm, and prevention of bronchiolitis obliterans. Therefore, it is mandatory to monitor vigilantly the patient and to treat aggressively the disease [8,16].

## 13. Conclusions

Because of its various clinical features, PNP represents a challenge for the clinician. In order to detect PNP earlier and to treat patients better, a cooperation between dermatologists, oncologists, otolaryngologists, ophthalmologists, and surgeons is recommended. Although different immunological markers have been found, the pathogenesis of PNP is still unknown. Management of the underlying tumor is of paramount importance. However, several therapies have been attempted to treat this potentially lethal condition.

## Figures and Tables

**Table 1:** Diagnostic criteria [1,12,75].

Parameter	Criterion
**Clinical features**	Painful erosions involving mucosae with or without a multiform skin eruption producing blisters and erosions, occurring in association with an occult or evident neoplasm
**Histopathology**	Suprabasal intraepithelial acantholysis, vacuolar interface changes, necrosis of individual keratinocytes, and/or lichenoid inflammation
**Direct immunofluorescence**	Combined presence of IgG and complement (C3) granular-linear deposition within the epidermal intercellular spaces and along the basement-membrane zone
**Indirect immunofluorescence**	Presence of circulating antibodies that target the intercellular zone of stratified squamous or transitional epithelia
**Immunoprecipitation**	Typical complex of proteins, including desmoplakin I (250 kD), bullous pemphigoid antigen (230 kD), envoplakin (210 kD), desmoplakin II (210 kD), periplakin (190 kD) and α-2-macroglobulin-like-1 (170 kD)

**Table 2:** Resume of PNP diagnostic algorithm.

CLINIC	Bullous Lesions on Skin and Mucous Membranes	
PATHOLOGY	Acantholysis (intraepidermal bulla)	Sub-epidermal cleavage
DIF	Combined presence of IgG and complement (C3) granular-linear deposition within the epidermal intercellular spaces and along the basement-membrane zone	
IIF	Presence of circulating antibodies that target the intercellular zone of stratified squamous or transitional epithelia	
LABORATORY	AAB directed to several proteins, including desmoplakin I (250 kD), bullous pemphigoid antigen (230 kD), envoplakin (210 kD), desmoplakin II (210 kD), periplakin (190 kD) and α-2-macroglobulin-like-1 (170 kD) IgG anti-DSG 1 and 3 IgG anti-DSG 1 IgA anti-DSC	
DIAGNOSIS	PNP PV PF IgAP	Exclusion of PNP

**Abbreviations:** PNP Paraneoplastic pemphigus; DIF Direct immunofluorescence; IIF Indirect immunofluorescence; AAB Autoantibodies; PV Pemphigus vulgaris; PF Pemphigus foliaceus; IgAP IgA pemphigus.

**Table 3:** Differential diagnosis.

Differential Diagnosis
Pemphigus vulgaris Bullous pemphigoid Major aphthous stomatitis Oral lichen planus Lichen planus Drug eruption Erythema multiforme GVHD Stevens–Johnson syndrome Toxic epidermal necrolysis
